# CMV infection, CD19^+^ B cell depletion, and Lymphopenia as predictors for unexpected admission in the institutionalized elderly

**DOI:** 10.1186/s12979-021-00233-0

**Published:** 2021-05-04

**Authors:** Liang-Yu Chen, An-Chun Hwang, Chung-Yu Huang, Liang-Kung Chen, Fu-Der Wang, Yu-Jiun Chan

**Affiliations:** 1 Institute of Public Health, National Yang Ming Chiao Tung University, No. 155, Sec. 2, Li-Nong St, Taipei, 11221 Taiwan; 2 Aging and Health Research Center, National Yang Ming Chiao Tung University, No. 155, Sec. 2, Li-Nong St, Taipei, 11221 Taiwan; 3 Center for Geriatrics and Gerontology, Taipei Veterans General Hospital, No. 201, Sec. 2, Shi-Pai Rd, Taipei, 11217 Taiwan; 4 School of Medicine, National Yang Ming Chiao Tung University, No. 155, Sec. 2, Li-Nong St, Taipei, 11221 Taiwan; 5 Division of Infectious Disease, Department of Medicine, Taipei Veterans General Hospital, No. 201, Sec. 2, Shi-Pai Rd, Taipei, 11217 Taiwan; 6 Division of Microbiology, Department of Pathology and Laboratory Medicine, Taipei Veterans General Hospital, No. 201, Sec. 2, Shi-Pai Rd, Taipei, 11217 Taiwan

**Keywords:** CD3^+^, CD19^+^, Chronic infection, Cytomegalovirus, The elderly, Immunosenescence, Lymphopenia

## Abstract

**Background:**

Chronic infections played a detrimental role on health outcomes in the aged population, and had complex associations with lymphocyte subsets distribution. Our study aimed to explore the predictive roles of chronic infections, lymphopenia, and lymphocyte subsets on unexpected admission and mortality in the institutionalized oldest-old during 3 year follow-up period.

**Results:**

There were 163 participants enrolled prospectively with median age of 87.3 years (IQR: 83.1–90.2), male of 88.3%, and being followed for 156.4 weeks (IQR: 136.9–156.4 weeks). The unexpected admission and mortality rates were 55.2 and 24.5% respectively. The Cox proportional hazards models demonstrated the 3rd quartile of cytomegalovirus IgG (OR: 3.26, 95% CI: 1.55–6.84), lymphopenia (OR: 2.85, 95% CI: 1.2–6.74), and 1st quartile of CD19^+^ B cell count (OR: 2.84, 95% CI: 1.29–6.25) predicted elevated risks of unexpected admission after adjusting for potential confounders; while the 3rd quartile of CD3^+^ T cell indicated a reduced risk of mortality (OR: 0.19, 95% CI: 0.05–0.71). Negative association between CMV IgG and CD19^+^ B cell count suggested that CMV infection might lead to B cell depletion via decreasing memory B cells repertoire.

**Conclusions:**

CMV infection, lymphopenia, and CD19^+^ B cell depletion might predict greater risk of unexpected admission, while more CD3^+^ T cell would suggest a reduced risk of mortality among the oldest-old population. A non-linear or U-shaped relationship was supposed between health outcomes and CMV infection, CD3^+^ T cell, or CD19^+^ B cell counts. Further prospective studies with more participants included would be needed to elucidate above findings.

## Background

In contrast to acute infections that trigger pro-inflammatory cytokine storms, chronic infections indicate an unique status of dynamic equilibrium between pathogens replication and host immune response [1]. The global prevalence of chronic infections estimated > 90% for Varicella zoster virus (VZV), 83% for cytomegalovirus (CMV), 5% for hepatitis B virus (HBV), and 2.5% for hepatitis C virus (HCV), and the prevalence is expected to be higher for HBV and HCV in Asia-Pacific region [1–3]. The majority of chronic infections are believed to be harmless that cause merely subclinical illness in immunocompetent hosts, but opportunistic clinical diseases among immunocompromised persons, congenital abnormalities in neonates, or tumor growth in specific target organs due to intrinsic oncogenicity [1, 2].

Accumulating evidence suggests a detrimental role of chronic infection on physical function, cognition, and other health outcomes in immunocompetent populations, especially in the older population. Chronic infection also had a positive association with geriatric syndromes among the elderly. The Northern Manhattan Study reported that chronic infection burden had a negative impact on cognitive performance, predicted accelerated cognitive decline, dementia onset, and stroke incidence [4]. Chronic herpesviral and *Chlamydia pneumoniae* (*C. pneumoniae*) infections were also reported as risk factors of Alzheimer’s disease and cardiovascular mortality [5, 6]. Moreover, CMV infection was proven a key indicator for cognitive impairment, frailty, also fatality in the Women’ Health and Aging studies I and II (WHAS I & II), the third National Health and Nutrition Examination Survey (NHANES III), and other prospective cohorts in the aged population [7–9]. Other than seropositivity, elevated CMV immunoglobulin (IgG) was considered as a biomarker for increased physical vulnerability and dysregulatory immunomodulation that driven by repetitive antigen exposure from reactivation of latent infection [10].

Adaptive immunity and lymphocyte subset distribution have complex interaction with physiological aging and chronic infection [11]. Absolute depletion in total lymphocyte count, or lymphopenia, predicted acute infection and mortality among the elderly and was taken an indicator for impaired adaptive immunity in aging process [12, 13]. While reduced CD19^+^ B cell count was reported as one parameter of “immune risk profile” along with poor T cell mitogenicity, accumulated CD8^+^ T cell count, inverted CD4^+^/CD8^+^, and CMV seropositivity, that predicted mortality and adverse health outcomes in the Swedish OCTO/NONA immune longitudinal studies in older persons [10]. However, the influence on lymphocyte subset distribution by chronic infection showed a great variation between different pathogens. Depletion in CD8^+^ and sometimes in CD4^+^ T cells by apoptotic pathway, called “T cell exhaustion”, were reported in HBV, HCV, and human immunodeficiency virus infection [11, 14]. In contrast, clonal expansion of antigen-specific CD8^+^ T cell by repetitive antigen stimulation, called “T cell senescence”, was disclosed in CMV infection [11, 15]. Reduced CD19^+^ B cell had been reported among middle-aged males with positive CMV infection, but the association still remained inconclusive [16].

Although chronic infection was frequently associated with adverse health outcomes in the elderly, the results based on accumulating evidences still remained controversial. The possible influence on geriatric syndromes, and complex interactions with lymphocyte subset distribution by chronic infection was not fully explored. Moreover, being more prevalent in Asia-Pacific region, the potential influence of HBV and HCV infections in the older generation was not yet investigated. The Veteran Affairs - Comprehensive Geriatric Assessment (VA-CGA) study in Taiwan was initiated since 2012, enrolled residents receiving elder care and assist-living care in the retirement communities for the veterans, and provided periodic assessment of geriatric syndromes by well-trained research staff every 6 months [17]. Residents were basically physically independent, cognitively intact, had less social engagement, and experienced common environmental exposures after allocation. Extended research by serological tests on chronic infection including CMV, VZV, HBV, HCV, and *C. pneumoniae* was started since 2016 among residents receiving elder care.

Thus, our study aimed to answer the possible impacts of chronic infections, lymphopenia, and lymphocyte subsets on unexpected admission and mortality in 3-year follow-up period among the older population at the retirement communities for veterans, as well as the association between chronic infection and lymphocyte subset distribution.

## Results

There were 163 participants enrolled with a median age of 87.3 years (IQR: 83.1–90.2 years), male of 88.3%, and being followed for a median of 156.4 weeks (IQR: 136.9–156.4 weeks). The physical function and cognitive status were relatively intact among participants, with median Barthel index score of 95 (IQR: 90–100) and Mini-Mental State Examination (MMSE) score of 26 (IQR: 23–29). Seropositivity was 99.4% of CMV (median IgG 236.8 IU/mL, IQR: 174.1–722.1 IU/mL), 92% of VZV (median IgG 3.46 S/CO, IQR: 2.51–4.7 S/CO), 57.1% of HBV surface antigen (HBsAg) (median IgG 17 mIU/mL, IQR: 3.8–50.8 mIU/mL), 7.4% of HCV (median IgG 0.07 S/CO, IQR: 0.05–0.1 S/CO), and 84.7% of *C. pneumoniae* (median IgG 1.49 S/CO, IQR: 1.28–1.63 S/CO) respectively. Five 5 participants (3.1%) moved out the retirement communities without registration of final health outcome. The unexpected admission rate was 55.2% (57.7% by infectious disease, 12.2% by cardiovascular disease, and 30% by others including traumatic injury, acute delirium, acute kidney injury, etc.), and the 3-year mortality rate was 24.5% (37.5% by infectious diseases, 17.5% by cardiovascular disease, and 45% by others including malignancy, brainstem hemorrhage, acute gastrointestinal bleeding, etc.). Demographic characteristics, chronic illness, geriatric syndromes, laboratory parameters in details were listed in Table 1.

**Table 1:** Baseline demographic characteristics of participants

Demographic and clinical characteristics	Participants ***N*** = 163 (100%)
**Age, years**	87.3 (83.1–90.2)
**Male gender (%)**	144 (88.3%)
**Follow-up duration, weeks**	156.4 (136.9–156.4)
**Move out from retirement communities**	5 (3.1%)
**Unexpected admission rate (%)**	90 (55.2%)
Infectious diseases-related (%)	52 (57.7%)
Cardiovascular diseases-related (%)	11 (12.2%)
Others (%)	27 (30%)
**Mortality rate (%)**	40 (24.5%)
Infectious diseases-related (%)	15 (37.5%)
Cardiovascular diseases-related (%)	7 (17.5%)
Others	18 (45%)
**Body mass index, kgs/m**^**2**^	24.2 (21.1–26)
**Cigarette Smoking**	
Ex-smoker (%)	17 (10.4%)
Active smoker (%)	20 (12.3%)
**Alcohol consumption**	
Ex-drinker (%)	10 (6.1%)
Active drinker (%)	28 (17.2%)
**Education**	
< 6 years (%)	53 (32.5%)
6–9 years (%)	51 (31.3%)
> 9 years (%)	48 (29.4%)
**Flu vaccine uptake (%)**	109 (66.9%)
**Underlying diseases**	
Cerebrovascular disease (%)	9 (5.5%)
Chronic liver disease (%)	2 (1.2%)
Chronic kidney disease (%)	4 (2.5%)
Chronic lung disease (%)	24 (14.7%)
Congestive heart failure (%)	7 (4.3%)
Dementia (%)	7 (4.3%)
Depression (%)	8 (4.9%)
Diabetes mellitus (%)	45 (27.6%)
Hypertension (%)	118 (72.4%)
Peptic ulcer disease (%)	26 (16%)
Peripheral arterial disease (%)	32 (19.6%)
Malignancy (%)	11 (6.7%)
**Charlson comorbidity index**	1 (0–2)
**Geriatric syndromes**	
Physical function by BI score	95 (90–100)
Risk of falls by JHFRAT score	12 (10–16)
Polypharmacy (%)	83 (50.9%)
Types of medicine	5 (3–6)
Cognition by MMSE score	26 (23–29)
Depression by GDS-5 score	0 (0–1)
Malnutrition by MUST score	0 (0–0)
Visual impairment (%)	52 (31.9%)
Hearing impairment (%)	27 (16.6%)
Sleep disorder (%)	46 (28.2%)
Use of hypnotic agents (%)	36 (22.1%)
Constipation (%)	44 (27%)
Stool incontinence (%)	3 (1.8%)
Urinary incontinence (%)	5 (3.1%)
**Laboratory tests**	
White blood cell count, cells/cumm	5900 (4900- 6700)
Hemoglobin, gm/dL	12.7 (11.6–13.6)
Platelet count, ×1,000 cells/cumm	180.5 (148.7–221)
Neutrophil count, cells/cumm	3157 (2472- 3914)
Lymphocyte count, cells/cumm	1800 (1500- 2300)
CD3^+^ T cell count, cells/cumm	1136 (903–1526)
CD4^+^ T cell count, cells/cumm	718 (557–926)
CD8^+^ T cell count, cells/cumm	390 (262–576)
CD19^+^ B cell count, cells/cumm	135 (81–205)
CD4^+^/CD8^+^	1.81 (1.25–2.54)
Albumin, gm/dL	4 (3.8–4.2)
Cholesterol, mg/dL	163 (141–188)
Triglyceride, mg/dL	87 (62–122)
HDL-C, mg/dL	48 (36–57)
LDL-C, mg/dL	97 (77–117)
BUN, mg/dL	19.5 (16–25)
Serum creatinine, mg/dL	0.96 (0.79–1.24)
Aspartate amonitransferase, U/L	21 (17–25)
hs-CRP, mg/dL	0.14 (0.08–0.32)
Lymphopenia (%)	12 (7.4%)
CD4^+^/CD8^+^ < 1	18 (11%)
**Chronic infection**	
CMV seropositivity (%)	162 (99.4%)
VZV seropositivity (%)	150 (92%)
HBsAg seropositivity (%)	93 (57.1%)
HCV seropositivity (%)	12 (7.4%)
*Chlamydia pneumoniae* seropositivity (%)	138 (84.7%)
CMV IgG, IU/mL	236.8 (174.1–722.1)
VZV IgG, S/CO	3.46 (2.51–4.7)
HBsAg IgG, mIU/mL	17 (3.8–50.8)
HCV IgG, S/CO	0.07 (0.05–0.1)
*Chlamydia pneumoniae* IgG, S/CO	1.49 (1.28–1.63)

Abbreviations: *BI* Barthel index, *CD* cluster of differentiation, *CMV* Cytomegalovirus, *GDS-5* Geriatric Depression Scale-5 items, *HBsAg* hepatitis B virus surface antigen, *HCV* hepatitis C virus, *HDL-C* high density lipoprotein-cholesterol, *hs-CRP* highly sensitive C reactive protein, *IgG* Immunoglobulin G, *JHFRAT* John Hopkins Fall Risk Assessment Tool, *LDL-C* Low density lipoprotein-cholesterol, *MMSE* Mini-Mental Status Examination, *MUST* Malnutrition Universal Screening Test, *VZV* Varicella zoster virus

In the Cox proportional hazards model, lymphopenia was shown an important predictor for both unexpected admission (OR: 2.97, 95% CI: 1.51–5.82) and mortality (OR: 2.32, 95% CI: 1–5.33) in model 1, remained significant for unexpected admission (OR: 2.85, 95% CI: 1.2–6.74) but less significant for mortality (OR: 1.9, 95% CI: 0.66–5.4) after adjustment for competing risk factors of mortality in model 2. The 3rd quartile of CMV IgG (OR: 3.26, 95% CI: 1.55–5.2) and the 1st quartile of CD19^+^ B cell count (OR: 2.84, 95% CI: 1.29–6.25) indicated greater risk of unexpected admission even after adjustment of competing risk factors of mortality in model 2, while the 3rd quartile of CD3^+^ T cell count had a reduced risk of mortality (OR: 0.19, 95% CI: 0.05–0.71) (Table 2). Due to uneven distribution in gender among participants, we performed another Cox regression surviving analysis including only males as model 2. The 3rd (OR: 3.76, 95% CI: 1.75–8.0), 4th (OR: 2.26, 95% CI: 1.07–4.76) quartile of CMV IgG, lymphopenia (OR: 2.8, 95% CI: 1.18–6.61), the 1st quartile of CD19+ B cell count (OR: 2.8, 95% CI: 1.24–6.29) still suggested greater risk of unexpected admission, and the 3rd quartile of CD3+ T cell count (OR: 0.19, 95% CI: 0.05–0.71) had a protective effect against mortality.

**Table 2:** The Cox proportional hazards model for mortality and unexpected admission

Variables	Unexpected admission: OR (95% CI)			Mortality: OR (95% CI)		
	Unadjusted	Model 1	Model 2	Unadjusted	Model 1	Model 2
**CMV IgG, IU/mL**						
< 174.1	1	1	1	1	1	1
174.1–236.8	1.23 (0.63, 2.39)	1.24 (0.62, 2.47)	2.05 (0.93, 4.51)	1.31 (0.52, 3.34)	1.37 (0.53, 3.5)	1.63 (0.57, 4.67)
236.8–722.1	2.56 (1.41, 4.64)**	2.84 (1.56, 5.2)**	3.26 (1.55, 6.84)**	1.36 (0.54, 3.38)	1.15 (0.45, 2.89)	0.92 (0.31, 2.66)
> 722.1	1.65 (0.88, 3.12)	1.7 (0.89, 3.24)	2.02 (0.97, 4.18)	1.49 (0.6, 3.71)	1.17 (0.46, 2.95)	0.97 (0.35, 2.67)
**HCV IgG, S/CO**						
< 0.0575	1	1	1	1	1	1
0.0575–0.07	0.85 (0.45, 1.58)	0.96 (0.51, 1.83)	1.1 (0.5, 2.42)	0.41 (0.17–0.97)*	0.51 (0.21, 1.26)	0.53 (0.18, 1.53)
0.07–0.1	1.03 (0.55–1.91)	1.39 (0.72, 2.67)	1.62 (0.77, 3.39)	0.58 (0.25, 1.32)	0.85 (0.35, 2.06)	0.95 (0.34, 2.58)
> 0.1	1.63 (0.91, 2.91)	1.71 (0.92, 3.18)	1.84 (0.91, 3.72)	0.53 (0.22, 1.26)	0.65 (0.26, 1.59)	0.65 (0.23, 1.81)
**Lymphopenia**	3.43 (1.8, 6.5)***	2.97 (1.51, 5.82)**	2.85 (1.2, 6.74)**	2.91 (1.29, 6.6)*	2.32 (1, 5.33)*	1.9 (0.66, 5.4)
**CD3**^**+**^ **T cell count, cells/cumm**						
> 1526	1	1	1	1	1	1
1136- 1526	0.97 (0.5, 1.88)	0.77 (0.38, 1.53)	0.43 (0.18, 1)	0.43 (0.13, 1.4)	0.21 (0.06, 0.73)*	0.19 (0.05, 0.71)*
903–1136	1.42 (0.77, 2.65)	0.98 (0.49, 1.92)	0.72 (0.34, 1.54)	1.12 (0.45, 2.76)	0.5 (0.19, 1.33)	0.59 (0.2, 1.73)
< 903	2.22 (1.23, 4.02)**	1.54 (0.78, 3.03)	1.04 (0.47, 2.28)	2.07 (0.91, 4.7)	1 (0.4, 2.47)	0.94 (0.33, 2.7)
**CD4**^**+**^ **T cell count, cells/cumm**						
> 926	1	1	1	1	1	1
718–926	1.34 (0.66, 2.69)	1.06 (0.51, 2.22)	0.63 (0.27, 1.46)	1.45 (0.46, 4.58)	1 (0.31, 3.18)	0.95 (0.27, 3.38)
557–718	2.15 (1.13, 4.11)*	1.55 (0.77, 3.12)	1.11 (0.52, 2.37)	2.23 (0.77, 6.42)	1.24 (0.42, 3.68)	1.31 (0.41, 4.13)
< 557	2.78 (1.46, 5.3)**	1.75 (0.84, 3.67)	1.57 (0.71, 3.46)	3.88 (1.42, 10.61)**	2.15 (0.75, 6.14)	2.55 (0.8, 8.06)
**CD19**^**+**^ **B cell count, cells/cumm**						
> 205	1	1	1	1	1	1
135–205	2.07 (1.06, 4.04)*	1.76 (0.86, 3.6)	1.59 (0.73, 3.46)	1.46 (0.46, 4.62)	0.97 (0.3, 3.12)	0.99 (0.28, 3.5)
81–135	1.54 (0.77, 3.08)	1.24 (0.57, 2.71)	1.05 (0.45, 2.48)	2.42 (0.84, 6.98)	1.35 (0.44, 4.12)	1.11 (0.34, 3.64)
< 81	3.1 (1.64, 5.84)***	2.24 (1.09, 4.63)*	2.84 (1.29, 6.25)**	3.67 (1.34, 10.03)*	1.99 (0.68, 5.81)	2.41 (0.79, 7.36)

*p < 0.05, **p < 0.01, ***p < 0.001

Model 1: Adjusted by age, gender, body mass index, highly-sensitive C-reactive protein, and multimorbidity by Charlson comorbidity index

Model 2: Adjusted by education status, cigarette smoking, alcohol consumption, albumin, low density lipoprotein cholesterol, medical history of acute myocardial ischemia, congestive heart failure, cerebrovascular accident, and diabetes mellitus, in addition to age, gender, body mass index, and multimorbidity

The CMV IgG positively correlated with age, flu vaccine uptake, risks of fall, usage or hypnotic agents, *C. pneumoniae* IgG, while negatively associated with education, cognition, and CD19^+^ B cell count (Table 3). For all lymphocyte, CD3^+^ T cell, and CD19^+^ B cell counts, there were positive associations with female gender, body mass index (BMI), cognitive performance, hemoglobin, albumin, cholesterol, low density lipoprotein-cholesterol, while negative correlations with age and flu vaccine uptake. Other results of Spearman correlation analysis were listed in Table 3.

**Table 3:** Results of Spearman correlation analysis between baseline characteristics

Indicators	Variables	Correlation coefficient	*p* value
CMV IgG	Age	0.19	0.017*
	Education	− 0.17	0.036*
	Flu vaccine uptake	0.278	0.001**
	JHFRAT score	0.219	0.006**
	MMSE score	−0.197	0.013*
	Usage of hypnotic agents	0.169	0.043*
	CD19^+^ B cell count	−0.179	0.026*
	*Chlamydia pneumoniae* IgG	0.194	0.015*
Lymphocyte count	Age	−0.291	< 0.001***
	Female Gender	0.226	0.004**
	Body mass index	0.296	< 0.001***
	Flu vaccine uptake	−0.192	0.017*
	Smoking history	−0.194	0.014*
	Diabetes mellitus	0.18	0.024*
	JHFRAT	−0.225	0.004**
	MMSE score	0.227	0.004**
	MUST score	−0.259	0.001**
	White blood cell count	0.5	< 0.001***
	Hemoglobin	0.261	0.001**
	Platelet count	0.185	0.018*
	CD3^+^ T cell count	0.862	< 0.001***
	CD4^+^ T cell count	0.757	< 0.001***
	CD8^+^ T cell count	0.632	< 0.001***
	CD19^+^ B cell count	0.449	< 0.001***
	Albumin	0.297	< 0.001***
	Cholesterol	0.319	< 0.001***
	Triglyceride	0.177	0.024*
	LDL-C	0.234	0.003**
	hs-CRP	−0.186	0.018*
CD3^+^ T cell count	Age	−0.337	< 0.001***
	Female Gender	0.304	< 0.001***
	Body mass index	0.328	< 0.001***
	Flu vaccine uptake	−0.209	0.01*
	Diabetes mellitus	0.195	0.015*
	JHFRAT score	−0.168	0.035*
	MMSE score	0.193	0.015*
	MUST score	−0.224	0.005
	White blood cell count	0.401	< 0.001***
	Hemoglobin	0.263	0.001**
	Platelet count	0.17	0.032*
	Lymphocyte count	0.862	< 0.001***
	CD4^+^ T cell count	0.835	< 0.001***
	CD8^+^ T cell count	0.792	< 0.001***
	CD19^+^ B cell count	0.423	< 0.001***
	Albumin	0.239	0.003**
	Cholesterol	0.364	< 0.001***
	LDL-C	0.281	< 0.001***
	Triglyceride	0.206	0.01*
	*Chlamydia pneumoniae* IgG	−0.17	0.033*
CD19^+^ B cell count	Age	−0.471	< 0.001***
	Female gender	0.337	< 0.001***
	Body mass index	0.265	0.001**
	Flu vaccine uptake	−0.278	0.001**
	Alcohol consumption	0.215	0.007**
	Chronic liver disease	0.184	0.02*
	Barthel index score	0.23	0.004**
	MMSE score	0.24	0.002**
	White blood cell count	0.202	0.011*
	Hemoglobin	0.232	0.003**
	Lymphocyte count	0.449	< 0.001***
	CD3^+^ T cell count	0.423	< 0.001***
	CD4^+^ T cell count	0.587	< 0.001***
	CD4^+^/CD8^+^	0.343	< 0.001***
	Albumin	0.192	0.016*
	Cholesterol	0.299	< 0.001***
	LDL-C	0.258	0.001**
	Creatinine	−0.168	0.035*
	CMV IgG	−0.179	0.026*

*p < 0.05, **p < 0.01, ***p < 0.001

Abbreviations: *CD* cluster of differentiation, *CMV* cytomegalovirus, *HDL-C* high density lipoprotein-cholesterol, *hs-CRP* highly sensitive C reactive protein, *IgG* Immunoglobulin G, *JHFRAT* John Hopkins Fall Risk Assessment Tool, *LDL-C* Low density lipoprotein-cholesterol, *MMSE* Mini-Mental Status Examination, *MUST* Malnutrition Universal Screening Test, *VZV* Varicella zoster virus

Additional adjustment for associating factors was performed in final model and still reported an elevated risk of unexpected admission for the 3rd quartile of CMV IgG (OR: 2.66, 95% CI: 1.14–6.2). With adjustment for correlating factors after removing white blood cell count, lymphocyte and subset counts, lymphopenia still indicated an increased risk of unexpected admission (OR: 3.59, 95% CI: 1.34–9.59), and the 3rd quartile of CD3^+^ T cell remained a reduced risk of mortality (OR: 0.38, 95% CI: 0.15–0.95).

## Discussion

Comparing with other chronic infections, CMV infection was a potential predictor for unexpected admission but not mortality, and the 3rd quartile of CMV IgG suggested an elevated risk of unexpected admission among the oldest-old persons even after adjustment for competing risk factors of mortality. For the CMV seroprevalence of 99.4% in our participants and 91.7% of blood donors in Taiwan, the CMV seropositivity would not be a good biomarkers on outcomes prediction [18]. Increased frailty incidence had been disclosed in the elderly with the highest CMV IgG in WHAS I & II, as well as poor survival possibility and rising incidence of cardiovascular disease in the Sacramento Area Latino Study on Aging and the population-based European Prospective Investigation of Cancer- Norfolk cohort study [8, 19, 20]. On the other hand, the BELFRAIL and another cohort studies among the oldest-old population revealed a neutral role of CMV infection on frailty and mortality [21, 22]. A non-linear or U-shaped relationship was supposed between CMV infection and adverse health outcomes in the oldest-old population, which would lead to diverse findings between studies. To the best of our knowledge, this study would be the first one to adopt unexpected admission for outcome assessment, and to present a non-linear pattern of CMV infection on outcome prediction. High CMV seroprevalence in Asian population since their childhood might account for the difference from previous studies including mostly Caucasian population [23].

The bottom quartile of CD19^+^ B cell count showed a positive association with unexpected admission in 3-year follow-up duration, and the effect weaned when adjusted for CMV IgG in final model. When we created interaction term between CMV IgG and CD19^+^ B cell count, there was significant interaction found in Cox proportional hazard model. Even CMV infection was shown a better predictor for risk of unexpected admission, reduced CD19^+^ B cell count was believed to be the mediator for adverse health outcome. The paradoxically negative correlation was disclosed between the CMV IgG and CD19^+^ B cell count, which was supposed the CMV-driven memory B cell exhaustion as previous study in middle-aged males [16]. Depletion in CD19^+^ B cell had been recognized an important indicator of the “immune risk profile”, showed positive association with male gender, advanced age, and CMV infection [16, 24]. Lower CD19^+^ B cell count was reported an independent predictor of mortality in patients under hemodialysis, and was associated with frailty and poor physical function in the elder population [25–27]. However, due to limited publications available at present, more studies would be needed for approaching the undermining pathogenic mechanism of CMV infection on CD19^+^ B cell count and long-term health outcomes.

Lymphopenia was shown an important risk factor of unexpected admission but not of mortality in our participants. The NHANES study retrospectively recognized a positive association between lymphopenia and mortality of either cardiovascular or non-cardiovascular causes in the general population [12]. The Copenhagen General Population Study demonstrated lymphopenia as a risk factor of admission and mortality due to infectious disease prospectively [13]. There were also prolonged length of stay and elevated in-hospital mortality reported among the older inpatients with lymphopenia [28]. Even lymphopenia predicts infection incidence, it failed to indicate an increased risk of mortality in the institutionalized elderly in our previous study [29]. Variation in lymphocyte subset distributions might be the possible explanation for this equivocal findings on predicting mortality by lymphopenia [11].

Lowest risk of mortality in the elderly was reported in the 3rd quartile group of CD3^+^ T cell count in our participants even after adjustment for all correlating factors. There had been a prospective study revealing a higher CD3^+^, CD4^+^ T cell counts and CD4^+^/CD8^+^ among survivors in a 2-year follow-up in the healthy Chinese elderly [30]. But our study failed to discover the ability by CD4^+^, CD8^+^ T cell counts or CD4^+^/CD8^+^ on outcomes prediction in the 3-year period.

Several limitations were noticed in our study. First, the non-linear or U-shaped relationship between risk factors and health outcomes might relate to the baseline characteristics of study participants at enrollment. We had tried adjusting all possibly confounding factors of all-cause mortality as possible to increase the strength of study, but more studies with larger sample size were still needed to prove our findings. Even limited participants were enrolled, with minimal drop-out rate of 3.1% and a follow-up period of 3 years, we believed above findings were convincing enough for male elderly through adequate statistical methods. Second, the proinflammatory cytokines were not included at initial study design except highly sensitive-C reactive protein, thus the complex interactions between chronic infection, lymphocyte subsets, inflammation, and health outcomes were not fully evaluated. Final, the current categories by flow cytometry could not distinguish the naïve or well-differentiated cells from the same lymphocyte subsets. Further studies to explore the possible influence of naïve or well-differentiated cell distributions would be needed for answering more questions.

## Conclusions

CMV infection, lymphopenia, and CD19^+^ B cell depletion predicted greater risk of unexpected admission, while more CD3^+^ T cell would suggest a reduced risk of mortality among the oldest-old population at institutes. A non-linear or U-shaped relationship was supposed between health outcome and CMV infection, CD3^+^ T cell, or CD19^+^ B cell counts. Further prospective studies with more participants included would be needed to elucidate above findings.

## Methods

### Participants

This prospective study enrolled residents receiving elder care with relatively intact physical and cognitive function at three retirement communities for veterans in Taiwan from July 1st, 2016 to January 31st, 2017. Potential candidates were invited for participation if they fulfilled the following criteria, (a) age ≥ 65 years, (b) stayed at the retirement communities for more than 3 months, (c) free of acute illness 2 weeks prior to study enrollment, and (d) with life expectancy for ≥ 1 year. Participants were excluded for those who (a) could not complete the baseline assessment and laboratory tests, (b) would not like to be followed in study period, or (c) would request for study withdrawal. All participants received baseline anthropometric measurements, comprehensive geriatric assessment, and venous blood sampling at enrollment by well-trained staffs. Demographic characteristics, underlying diseases, and laboratory parameters were collected, while a composite score of Charlson Comorbidity Index (CCI) was calculated for adjustment [31].

### Measurements

#### Geriatric syndromes

Physical performance was approached by the Barthel index for activities of daily living. The higher Barthel index score suggests a better physical function [32]. Risk of falls was evaluated by Johns Hopkins Fall Risk Assessment Tool (JHFRAT) for home health care [33]. Scores of JHFRAT were calculated from established items, including (1) age, (2) fall history ≤6 months, (3) bowel and urine elimination, (4) usage of high risk medications, (5) patient care equipment, (6) mobility, and (7) cognition. The more JHFRAT score indicated elevated risks of fall. Polypharmacy was identified if residents concurrently took ≥5 types of medications [34]. Types of medications were counted and registered for analysis.

Cognition was assessed by the MMSE, Chinese version. The lower MMSE score indicates a poor cognition [35]. Depressive symptom was approached by the geriatric depression scale-5 item version (GDS-5). Participants with higher GDS-5 had more possibility for depression [36]. Risk of undernutrition was defined by Malnutrition Universal Screening Tool (MUST). Participants with higher MUST score faced more risks of undernutrition [37]. Visual impairment, hearing difficulty, sleep disorder, as well as urinary and stool incontinence were also recorded.

### Laboratory parameters

Peripheral venous blood samples were sampled via the antecubital vein in the morning after an overnight fasting. Complete blood counts and differential counts were analyzed by the automated cellular analysis system Beckman Coulter DxH 800 hematology analyzer (Beckman Coulter, Miami, FL). Biochemical parameters of albumin, lipid profile, high sensitive-C reactive protein (hs-CRP), fasting glucose, aspartate transaminase, blood urea nitrogen, and serum creatinine were measured by the ARCHITECT i2000SR immunoassay analyzer (Abbott Diagnostics, Abbott Park, IL, USA). Lymphopenia was defined by absolute lymphocyte count < 1000/cumm [29].

Lymphocyte subsets were determined flow-cytometric analysis system Beckman Coulter FC500 flow cytometer (Beckman Coulter, Miami, FL) in freshly drawn peripheral blood after adequate processing as the manufacturer’s instruction. Serum CMV IgG, VZV IgG, HBsAg IgG, HCV IgG, and *C. pneumoniae* IgG were measured by a chemiluminescent immunoassay on the ARCHITECT i2000SR immunoassay analyzer (Abbott Diagnostics, Abbott Park, IL, USA).

### Outcome measurement

Unexpected admission and mortality were recorded of each individual in 3-year follow-up period, and the main etiology of each event was also registered. For those who lost to follow-up in study period would be treated as censored cases, and time for follow-up were documented until the last available information.

### Statistical analysis

Results for data analysis are expressed as median with interquartile range (IQR) for continuous variables, and numbers (%) for categorical variables. Quartile grouping was performed on CMV IgG, VZV IgG, HBsAg IgG, HCV IgG, *C. pneumoniae* IgG, CD3^+^ T cell count, CD4^+^ T cell count, CD8^+^ T cell count, CD19^+^ B cell count, and CD4^+^/CD8^+^. Cox proportional hazard model with bootstrapping for 1000 was used to assess the possible impacts on unexpected admission and mortality of lymphopenia and each quartile groups. Adjustment for age, gender, nutritional status by BMI, inflammatory status by hs-CRP, and multimorbidity by CCI was performed in model 1, while further adjustments for competing risk factors of all-cause mortality including education status, cigarette smoking, alcohol consumption, serum albumin, low density lipoprotein-cholesterol, congestive heart failure, cerebrovascular disease, and diabetes mellitus were performed in model 2. Non-parametric method of Spearman correlation analysis was used for comparison in case of possibly non-Gaussian distribution of numerical data. Another Cox proportional hazard model with additional adjustment for associated factors was performed finally. All data analyses were carried out with the Statistical Package for the Social Sciences for Windows version 20.0 (SPSS, Chicago, IL, USA), and variables were considered as statically significant if *P* < 0.05.

## Data Availability

All collected data and analyses during the current study are available from the corresponding author on reasonable request at email address: yjchan@vghtpe.gov.tw

## Abbreviations

CD: Cluster of differentiation
CMV: Cytomegalovirus
*C. pneumoniae*: *Chlamydia pneumoniae*
GDS-5: Geriatric Depression Scale-5 items
HBsAg: Hepatitis B virus surface antigen
HBV: Hepatitis B virus
HCV: Hepatitis C virus
IgG: Immunoglobulin G
hs-CRP: Highly sensitive-C reactive protein
JHFRAT: John Hopkins Fall Risk Assessment Tool
MMSE: Mini-Mental Status Examination
MUST: Malnutrition Universal Screening Test
NHANES: The National Health and Nutrition Examination Survey
VZV: Varicella zoster virus
WHAS: The Women’ Health and Aging studies

## References

[1] Virgin HW, Wherry EJ, Ahmed R (2009). Redefining chronic viral infection. Cell..

[2] Ringelhan M, McKeating JA, Protzer U (2017). Viral hepatitis and liver cancer. Philos Trans R Soc Lond Ser B Biol Sci.

[3] Zuhair M, Smit GSA, Wallis G, Jabbar F, Smith C, Devleesschauwer B, Griffiths P (2019). Estimation of the worldwide seroprevalence of cytomegalovirus: a systematic review and meta-analysis. Rev Med Virol.

[4] Zhao C, Strobino K, Moon YP, Cheung YK, Sacco RL, Stern Y, Elkind MSV (2020). APOE 4 modifies the relationship between infectious burden and poor cognition. Neurol Genet.

[5] Qin Q, Li Y (2019). Herpesviral infections and antimicrobial protection for Alzheimer's disease: implications for prevention and treatment. J Med Virol.

[6] Rupprecht HJ, Blankenberg S, Bickel C, Rippin G, Hafner G, Prellwitz W, Schlumberger W, Meyer J, AutoGene Investigators (2001). Impact of viral and bacterial infectious burden on long-term prognosis in patients with coronary artery disease. Circulation..

[7] Mendy A, Gasana J, Vieira ER, Diallo H (2014). Prospective study of cytomegalovirus seropositivity and risk of mortality from diabetes. Acta Diabetol.

[8] Wang GC, Kao WH, Murakami P, Xue QL, Chiou RB, Detrick B (2010). Cytomegalovirus infection and the risk of mortality and frailty in older women: a prospective observational cohort study. Am J Epidemiol.

[9] Barnes LL, Capuano AW, Aiello AE, Turner AD, Yolken RH, Torrey EF, Bennett DA (2015). Cytomegalovirus infection and risk of Alzheimer disease in older black and white individuals. J Infect Dis.

[10] Pawelec G (2018). Immune parameters associated with mortality in the elderly are context-dependent: lessons from Sweden, Holland and Belgium. Biogerontology.

[11] Minato N, Hattori M, Hamazaki Y (2020). Physiology and pathology of T-cell aging. Int Immunol.

[12] Zidar DA, Al-Kindi SG, Liu Y, Krieger NI, Perzynski AT, Osnard M (2019). Association of Lymphopenia with Risk of mortality among adults in the US general population. JAMA Netw Open.

[13] Warny M, Helby J, Nordestgaard BG, Birgens H, Bojesen SE (2018). Lymphopenia and risk of infection and infection-related death in 98,344 individuals from a prospective Danish population-based study. PLoS Med.

[14] Saeidi A, Zandi K, Cheok YY, Saeidi H, Wong WF, Lee CYQ, Cheong HC, Yong YK, Larsson M, Shankar EM (2018). T-cell exhaustion in chronic infections: reversing the state of exhaustion and reinvigorating optimal protective immune responses. Front Immunol.

[15] Bektas A, Schurman SH, Sen R, Ferrucci L (2017). Human T cell immunosenescence and inflammation in aging. J Leukoc Biol.

[16] van der Heiden M, van Zelm MC, Bartol SJW, de Rond LGH, Berbers GAM, Boots AMH, Buisman AM (2016). Differential effects of Cytomegalovirus carriage on the immune phenotype of middle-aged males and females. Sci Rep.

[17] Chen LY, Wu YH, Huang CY, Liu LK, Hwang AC, Peng LN, Lin MH, Chen LK (2017). Predictive factors for dementia and cognitive impairment among residents living in the veterans' retirement communities in Taiwan: implications for cognitive health promotion activities. Geriatr Gerontol Int.

[18] Lu SC, Chin LT, Wu FM, Hsieh GJ, Haung SP, Chen JC, Chang AC, Hsieh WK, Chen BH (1999). Seroprevalence of CMV antibodies in a blood donor population and premature neonates in the south-Central Taiwan. Kaohsiung J Med Sci.

[19] Gkrania-Klotsas E, Langenberg C, Sharp SJ, Luben R, Khaw KT, Wareham NJ (2012). Higher immunoglobulin G antibody levels against cytomegalovirus are associated with incident ischemic heart disease in the population-based EPIC-Norfolk cohort. J Infect Dis.

[20] Gkrania-Klotsas E, Langenberg C, Sharp SJ, Luben R, Khaw KT, Wareham NJ (2013). Seropositivity and higher immunoglobulin g antibody levels against cytomegalovirus are associated with mortality in the population-based European prospective investigation of Cancer-Norfolk cohort. Clin Infect Dis.

[21] Mathei C, Vaes B, Wallemacq P, Degryse J (2011). Associations between cytomegalovirus infection and functional impairment and frailty in the BELFRAIL cohort. J Am Geriatr Soc.

[22] Cao Dinh H, Bautmans I, Beyer I, Mets T, Onyema OO, Forti LN, Renmans W, Vander Meeren S, Jochmans K, Vermeiren S, Vella-Azzopardi R, Njemini R, Gerontopole Brussels Study Group (2019). Association between Immunosenescence phenotypes and pre-frailty in older subjects: does Cytomegalovirus play a role?. J Gerontol A Biol Sci Med Sci.

[23] Fang FQ, Fan QS, Yang ZJ, Peng YB, Zhang L, Mao KZ, Zhang Y, Ji YH (2009). Incidence of cytomegalovirus infection in Shanghai. China Clin Vaccine Immunol.

[24] Apoil PA, Puissant-Lubrano B, Congy-Jolivet N, Peres M, Tkaczuk J, Roubinet F, Blancher A (2017). Influence of age, sex and HCMV-serostatus on blood lymphocyte subpopulations in healthy adults. Cell Immunol.

[25] Yang FJ, Shu KH, Chen HY, Chen IY, Lay FY, Chuang YF, Wu CS, Tsai WC, Peng YS, Hsu SP, Chiang CK, Wang G, Chiu YL (2018). Anti-cytomegalovirus IgG antibody titer is positively associated with advanced T cell differentiation and coronary artery disease in end-stage renal disease. Immun Ageing.

[26] Marcos-Pérez D, Sánchez-Flores M, Maseda A, Lorenzo-López L, Millán-Calenti JC, Gostner JM, Fuchs D, Pásaro E, Laffon B, Valdiglesias V (2018). Frailty in older adults is associated with plasma concentrations of inflammatory mediators but not with lymphocyte subpopulations. Front Immunol.

[27] Valdiglesias V, Sánchez-Flores M, Maseda A, Marcos-Pérez D, Millán-Calenti JC, Pásaro E, Lorenzo-López L, Laffon B (2015). Lymphocyte subsets in a population of nonfrail elderly individuals. J Toxicol Environ Health A.

[28] Rubio-Rivas M, Formiga F, Grillo S, Gili F, Cabrera C, Corbella X (2016). Lymphopenia as prognostic factor for mortality and hospital length of stay for elderly hospitalized patients. Aging Clin Exp Res.

[29] Chang CJ, Chen LY, Liu LK, Lin MH, Peng LN, Chen LK (2014). Lymphopenia and poor performance status as major predictors for infections among residents in long-term care facilities (LTCFs): a prospective cohort study. Arch Gerontol Geriatr.

[30] Wang YH, Zhang JY, Qiao FF, Zhu J, Yin F, Han H (2012). Correlation between T lymphocyte subsets in peripheral blood lymphocytes and 2-year all-cause mortality in an apparently healthy elderly Chinese cohort. Chin Med J.

[31] Charlson ME, Pompei P, Ales KL, MacKenzie CR (1987). A new method of classifying prognostic comorbidity in longitudinal studies: development and validation. J Chronic Dis.

[32] Mahoney FI, Barthel DW (1965). Functional evaluation: the Barthel index. Md State Med J.

[33] Hnizdo S, Archuleta RA, Taylor B, Kim SC (2013). Validity and reliability of the modified John Hopkins fall risk assessment tool for elderly patients in home health care. Geriatr Nurs.

[34] Jorgensen T, Johansson S, Kennerfalk A, Wallander MA, Svardsudd K (2001). Prescription drug use, diagnoses, and healthcare utilization among the elderly. Ann Pharmacother.

[35] Creavin ST, Wisniewski S, Noel-Storr AH, Trevelyan CM, Hampton T, Rayment D, et al. Mini-Mental State Examination (MMSE) for the detection of dementia in clinically unevaluated people aged 65 and over in community and primary care populations. Cochrane Database Syst Rev. 2016:CD011145. 10.1002/14651858.CD011145.pub2 PMC8812342 26760674

[36] Hoyl MT, Alessi CA, Harker JO, Josephson KR, Pietruszka FM, Koelfgen M, Mervis JR, Fitten LJ, Rubenstein LZ (1999). Development and testing of a five-item version of the geriatric depression scale. J Am Geriatr Soc.

[37] Todorovic V, Russell C, Stratton R, Ward J, Elia N (2003). The ‘MUST’ Explanatory Booklet: A Guide to the ‘Mulnutrition Universal Screenung Tool’ (MUST) for Adults.

